# Endocardial Regulation of Cardiac Development

**DOI:** 10.3390/jcdd9050122

**Published:** 2022-04-19

**Authors:** Lara Feulner, Patrick Piet van Vliet, Michel Puceat, Gregor Andelfinger

**Affiliations:** 1 Cardiovascular Genetics, CHU Sainte-Justine Research Centre, Montreal, QC H3T 1C5, Canada; lara.michele.feulner@umontreal.ca (L.F.); patrick.van.vliet.hsj@ssss.gouv.qc.ca (P.P.v.V.); 2 Department of Molecular Biology, University of Montreal, Montreal, QC H3T 1J4, Canada; 3 LIA (International Associated Laboratory) CHU Sainte-Justine, Montreal, QC H3T 1C5, Canada; michel.puceat@inserm.fr; 4 LIA (International Associated Laboratory) INSERM, 13885 Marseille, France; 5 INSERM U-1251, Marseille Medical Genetics, Aix-Marseille University, 13885 Marseille, France; 6 Department of Biochemistry and Molecular Medicine, University of Montreal, Montreal, QC H3T 1J4, Canada; 7 Department of Pediatrics, University of Montreal, Montreal, QC H3T 1J4, Canada; 8 Department of Biochemistry, University of Montreal, Montreal, QC H3T 1J4, Canada

**Keywords:** endocardium, heart development, heart disease, BMP, Notch

## Abstract

The endocardium is a specialized form of endothelium that lines the inner side of the heart chambers and plays a crucial role in cardiac development. While comparatively less studied than other cardiac cell types, much progress has been made in understanding the regulation of and by the endocardium over the past two decades. In this review, we will summarize what is currently known regarding endocardial origin and development, the relationship between endocardium and other cardiac cell types, and the various lineages that endocardial cells derive from and contribute to. These processes are driven by key molecular mechanisms such as Notch and BMP signaling. These pathways in particular have been well studied, but other signaling pathways and mechanical cues also play important roles. Finally, we will touch on the contribution of stem cell modeling in combination with single cell sequencing and its potential translational impact for congenital heart defects such as bicuspid aortic valves and hypoplastic left heart syndrome. The detailed understanding of cellular and molecular processes in the endocardium will be vital to further develop representative stem cell-derived models for disease modeling and regenerative medicine in the future.

## 1. Introduction

The heart is the first organ to develop in vertebrates and originates from mesodermal cells in the anterior part of the primitive streak [1]. These cells migrate during gastrulation from the primitive streak to form the cardiac crescent, or first heart field (FHF) located in the splanchnic mesoderm underlying the head folds. The crescent fuses at the ventral midline and closes dorsally to form a primitive heart tube, which then elongates via contribution of cells from the second heart field (SHF) located posteromedially to the crescent. The early developing heart tube consists of an outer myocardial layer and an inner endocardial layer separated by an extracellular matrix (ECM) layer of cardiac jelly enriched in glycosaminoglycan proteins such as versican and hyaluronan [2,3]. The tube then undergoes rightward looping and segmentation into atria, ventricles, atrioventricular canal (AVC) and outflow tract (OFT). The atrioventricular (AV) cardiac valves separate the atria and ventricles, while semilunar (SL) valves separate the ventricles from the aorta and pulmonary artery. FHF cells contribute to most of the left ventricle (LV) and part of the atria, while SHF cells give rise to atria, the right ventricle (RV), OFT and inflow tract.

Normal myocardial patterning and communication between endocardium and myocardium is required for key developmental processes such as valvulogenesis. Valve development is initiated by swellings of cardiac jelly to form endocardial cushions, followed by inward migration of endocardial cells after their endothelial-to-mesenchymal transition (EndoMT) [4,5]. Another example is the formation of trabeculae, which are protrusions of cardiomyocytes from the inner wall of the ventricle that help maintain early blood flow. Trabeculation is initiated as endocardial cells penetrate the cardiac jelly in the ventricle to touchdown on myocardium. As endocardial ridges are generated between the touchdowns, a layer of the jelly becomes ECM bubbles, enclosing trabecular cardiomyocytes. Proliferation and growth of the trabecular unit toward the ventricular lumen then occurs [3].

In addition to its role in valvulogenesis and trabeculation, the endocardium is highly plastic and contributes to other tissues such as coronary vasculature. While historically less studied than myocardium, research over the last two decades has greatly enhanced our understanding of the endocardium and its role in normal and pathological development. In this review, we will summarize those findings and present current knowledge.

## 2. Development of the Endocardium

### 2.1. Endocardial Cell Origins

The contributing lineages and cell fate decisions leading to endocardial formation have been relatively challenging to elucidate, and two developmental models have been proposed. The pre-specification model proposes that cells in the primitive streak are pre-specified to become either myocardium or endocardium, prior to migration of their precursors. Avian and zebrafish studies have particularly supported this model. Introduction of beta-galactosidase expressing viral particles in chicks gave rise to clonal patches derived from a single infected precursor cell that contained either endocardium or myocardium, but not both [6,7]. Consistent with this, time-lapse microscopy and lineage tracing of zebrafish indicated that endocardium was derived from migrating hematopoietic and vascular lineage cells within the lateral plate mesoderm [8,9], arguing against a common endocardial-myocardial progenitor.

More recently, the multipotent model proposes that mesoderm in the cardiac crescent contains multipotent cells that can give rise to both endocardium and myocardium. Early mouse Cre-based fate mapping studies used cardiac lineage markers to label precursors that gave rise to both endocardium and myocardium [10,11,12,13]. For example, a *Mef2c* regulatory element driving Cre expression exclusively in the anterior heart field and its derivatives marks cells contributing both to endocardium in the RV and myocardium in the OFT [12].

In vitro studies also support multipotency, such as shown through induced differentiation of an embryonic stem cell (ESC) line with a brachyury-targeted GFP reporter. This drives a subset of GFP+ cells to become *FLK1* (aka *VEGFR2/KDR*)+ and form beating cTnT+ cells upon exposure to VEGF [14]. Transgenic Nuclear Factor of Activated T Cells 1 (NFATc1)-nuc-LacZ mice are capable of labeling endocardium, and ESCs can be further differentiated from this line. Wnt inhibition enhances myocardial and decreases endocardial differentiation, and vice versa upon Wnt3A addition. E-cadherin (ECD)^low^Flk1^low^ FACS-sorted cells express brachyury but not *Tal1* (aka *Scl*), suggesting nonvascular mesoderm; and only these cells show myocardial or endocardial differentiation following culture. ECD^low^Flk1^low^ cell-derived colonies express endocardial markers including *Nfatc1* and Neuregulin *(Nrg1*) while those from ECD^low^Flk1^high^ cells express hematopoietic markers such as *Bh1-globin*, suggesting that contrary to zebrafish, mouse endocardium is not specified from the mesoderm that generates the hemangioblast population [15]. However, a recent report showed that a population of *Tal1*-positive cells migrate from the distal border of the yolk sac and contribute to the endocardium at embryonic day (E)7.5 [16].

To help resolve endocardial origin models, Milgrom-Hoffmann et al. performed DiI mapping studies in chicks. Cells medial to the cardiac crescent gave rise to endocardium but not myocardium, and endothelial cells transplanted from stage 8 quails into the cardiac region of chick host embryos were able to contribute only to endocardium, demonstrating cell fate restriction. Lineage studies in mice suggested that the majority of the endocardium is derived from *Isl1*− endothelial lineage rather than *Isl+* SHF lineage. Furthermore, ablation of *Flk1* diminished endocardial cells in *Mesp1* and *Tie2* mouse lineages, and altered the proportion of *Isl1+* and *Isl1*− lineage-derived endocardial cells. This indicates that endocardial cells at least partly derive from vascular endothelial cells, which do not give rise to myocardium [17].

Lineage tracing of *Mesp1*, the earliest transcription factor (TF) to mark mesodermal cardiac precursor cells prior to the establishment of heart fields, in mice provides insight into very early endocardial specification. *Mesp1* labeling occurs at gastrulation, and *Smarcd3+* precursor cells at that stage are already assigned to specific regions of the heart, corresponding to FHF and SHF [18]. There does not appear to be a common progenitor for myocardial, endocardial or pericardial cells localized to the left and right ventricle, consistent with prior beta-galactosidase-based clonal studies of myocardium [19]. Importantly, while most cardiac progenitors became lineage-restricted at gastrulation, approximately 5% could contribute to multiple lineages including endocardium and myocardium [18]. Inducible *Mesp1* labeling in mice reveals that distinct populations of unipotent and bipotent cardiac progenitors can be identified at E6.25 and E7.25: early *Mesp1+* cells are unipotent epicardial-derived cell (EPDC) or FHF progenitors, the latter ones being able to differentiate either into cardiomyocytes (CM) or endothelial cells (EC). Late *Mesp1+* cells are all SHF progenitors, either unipotent (CM vs. EC) or bipotent (CM + smooth muscle, or CM + EC). This suggests that endocardium is mostly specified at gastrulation in mice, but some OFT and RV endocardium may originate from multipotent SHF precursors [20]. Consistent with these findings, a recent study combining genetic tracing of *T* and *Foxa2* expressing cells, single cell sequencing and live imaging indicated that the FHF and SHF of gastrulating mouse embryos subdivide early into distinct pools of cardiac progenitors. An orderly migration of myocardial progenitor cells from the primitive streak contributing first to the LV, then the RV, and finally, OFT and atria was seen [21].

**Figure 1:**
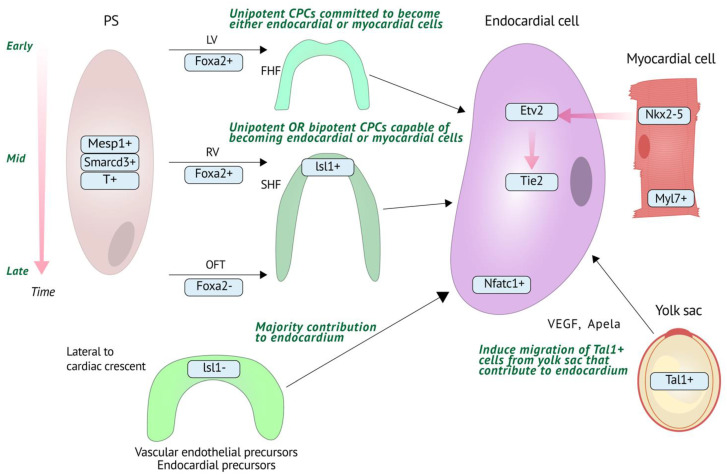
Schematic representation (not drawn to scale) of endocardial development. Unipotent cardiac progenitor cells (CPCs) committed to become either endocardial (*Etv2+*, *Nfatc1+*) or myocardial (*Nkx2-5+*, *Myl7+*) cells migrate from the primitive streak (PS) at an early stage to form the FHF, which contributes to the left ventricle (LV). Unipotent and bipotent (capable of becoming either endocardial or myocardial cells) CPCs migrate to form the *Isl+* SHF, which contributes to right ventricle (RV) and outflow tract (OFT) at mid-late PS stages, respectively. The contribution of *Isl+* SHF cells to endocardium is however small, as the majority of endocardium is derived from a population of *Isl1*− vascular endothelial progenitor cells located lateral to the cardiac crescent. The endocardium also receives a contribution from a population of *Tal1+* cells that migrate from the yolk sac at E7.5. Molecular mechanisms are illustrated on right side via red arrows in contrast to migration (black arrows). Endocardial *Etv2* is activated by NKX2-5 and *Tie2* is a downstream target of ETV2.

Discrepancies between the pre-specification and multipotent models were initially explained by the postulate that cardiogenic precursors retain plasticity within the cardiac crescent, but their fate decisions upon arrival in the crescent are constrained by positional cues within the embryo that are absent when culturing precursors ex vivo [22]. However, it is now clear that the pre-specification and multipotent endocardial precursor models together reflect how the endocardium as a whole is a heterogeneous mix of different lineages, which has clear implications for understanding the anatomical context of molecular mechanisms driving endocardial development and subsequent effects on other cardiac cell types. While some differences have been noted between mammalian and zebrafish endocardial development, the key processes appear to be conserved between species (Figure 1).

### 2.2. Molecular Regulation of Endocardial Development

The genetic pathways downstream of *Mesp1* governing endocardial development are still being elucidated. The TF ETV2 is particularly significant in this context given that *Etv2*-null mouse embryos lack endocardial and endothelial lineages [23]. This suggests that endocardium originates from *Etv2+* mesoderm, but whether ETV2 itself determines endocardial fate or simply confers competence to develop endocardium is still unclear [24]. A conserved NKX2-5 responsive element upstream of *Etv2* was identified, indicating that endocardial *Etv2* expression is at least in part regulated by myocardial NKX2-5 [23]. However, there is some *Nkx2-5* expression in endocardium [25]; so, a cell autonomous component to this regulation is possible. Interestingly, etv2 knockdown in zebrafish expressing an etv2-GFP construct resulted in some GFP+ cells becoming cardiomyocytes [26], suggesting ETV2 may play a role in myocardial versus endocardial lineage determination. Myocardial-specific, but not endocardial-specific, HAND2 also rescued impaired endocardial development in *hand2* mutant zebrafish [27].

The SRY-box TF SOX17, previously considered endoderm-specific, is also expressed in vascular endothelial cells, blood cells and heart endocardium [28]. *Sox17* ablation suppressed differentiation of mouse ESCs into cardiomyocytes in vitro [29]. Single cell sequencing revealed that *Sox17* is transiently expressed in 20–30% of NKX2-5+ mouse cardiac progenitor cells from E7.5–E8.5. Subsequent lineage tracing confirmed that *Sox17+* mesoderm cells give rise to endocardium. While gain-and-loss of function analyses suggested SOX17 is not required for endocardial fate, scRNA-seq of *Sox17* mutants showed transcriptomic misregulation in endocardial and myocardial cells impacting growth and proliferation [24].

The cues that regulate migration of endothelial precursors within the embryo are not fully understood yet. Single cell analysis of E7.25–E7.5 mouse embryos recently implicated Apela (aka Elabela or ELA) and VEGF as chemoattractants, regulating the migration of *Tal1+* cells contributing to endocardium [16]. Overall, further study is needed and anticipated in the future to fully detail the molecular pathways comprising regulation of endocardial development.

## 3. Endocardial Contribution to Cardiac Cell Types and Lineage-Specific Mechanisms

**Figure 2:**
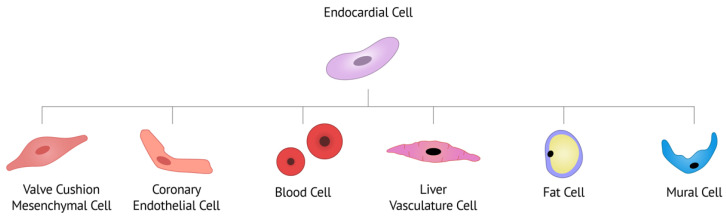
Known endocardial cell lineages, adapted from Zhang et al. [31]. Endocardial cells can develop into valve cushion mesenchymal cells (VICs), coronary endothelial cells, blood cells, liver vasculature cells, fat cells, and mural cells (potentially via “intermediate” cushion mesenchyme).

Endocardial cells have a high degree of plasticity and can develop into numerous cardiac cell types, including blood cells, valve cells, coronary endothelium, liver vasculature, fat cells and mural cells (Figure 2) [30,31]. The development of these distinct lineages often relies on similar overall mechanisms and signaling pathways, but with very specific anatomical context-dependent outcomes.

### 3.1. Hemogenic Endocardium

Embryonic endothelium serves both as a conduit for blood and as a source of hematopoietic progenitor stem cells [32]. In a model proposed by Van Handel et al., lateral plate mesoderm gives rise to either a “prospective hemogenic” lineage or an endocardial lineage, both of which could differentiate into cardiomyocytes. TAL1 establishes hemogenic endothelium and inhibits conversion of endocardium into cardiomyocytes [33]. A subsequent single cell analysis however did not find upregulation of cardiac markers in *Tal1^−/−^* endothelial cells, showing that TAL1 does not regulate endothelial versus cardiomyocyte fates [34].

Nakano et al. first identified a population of mouse hemogenic endocardial cells, after observing that early pre-circulation heart tube explants (1–5 somite stages) were capable of developing into erythroid and myeloid progenitors, and that CD41 (an early marker for hematopoietic progenitor cells) is expressed in a subset of endocardial cells. Lineage tracing revealed that the majority of those CD41+ cells arise from NKX2-5-derived endocardium. Further analysis suggested that NKX2-5+ endocardium and yolk sac endothelium give rise to hematopoietic progenitors via an NKX2-5 and ISL1-dependent mechanism, which contributes to circulating erythroid-myeloid cells until late gestation. Hemogenic endocardium may thus be derived from FLK1+/ISL1+/NKX2-5+ multipotent cardiac progenitors through endocardial intermediates [35].

Lineage tracing of NKX2-5+ and ISL1+ populations in embryonic chicks reveals that hemogenic endocardium originates from NKX2-5+ angioblasts in the primitive streak before migrating to the posterior lateral plate mesoderm and extraembryonic regions, and subsequently, into the endocardium and dorsal aorta [36]. Gain-of-function experiments showed that NKX2.5 induces mesodermal expression of hemo-angiogenic markers. Additional lineage tracing in mice demonstrated that NKX2-5 is expressed in extraembryonic tissue proximal to the cardiac crescent, and that NKX2-5+ cells make up a substantial contribution to RUNX1+/CD41+ hemogenic endothelium in the dorsal aorta and endocardium—thus providing in vivo evidence that NKX2-5+ progenitors contribute to hemogenic endocardium and that NKX2-5 has a role in initiating hemo-angiogenesis [36].

Interestingly, *Nfatc1-Cre* lineage tracing in mice identified hemogenic endocardium as a source of a subset of cardiac tissue macrophages during embryogenesis, with the caveat that non-endocardial sources for macrophages could not be fully excluded due to *Nfatc1* expression in yolk sac and liver [37]. Ablation of these endocardial-derived cardiac tissue macrophages (EcTMs) resulted in excessive mesenchymal cells in both aortic and mitral valves, suggesting that EcTMs are indispensable for valvular remodeling [37].

Recent work by Collart et al. showed that at least some of the endocardium, dorsal aorta and head vasculature in mice are derived from yolk sac cells. Given that haematopoiesis takes place in the latter two structures, they examined whether it also occurs in the endocardium. *Tal1* is strongly expressed in E8.5–E10.5 endocardium, which is notable since a high TAL1 threshold can result in cells becoming irreversibly committed to haematopoiesis. Furthermore, cells bud from the endocardium of transgenic E9–E9.5 *Tal1*-cerulean mouse hearts. Cells within the endocardium are *Flk1+*/*Tal1+* but detach from the endocardial wall in regions of lower FLK1. Given that haematopoietic cells lose *Flk1* expression, this indicates an endothelial-to-haematopoietic transition is taking place [16]. While Collart et al. did not further investigate these budding cells, these results are consistent with those of Nakano et al. [35].

### 3.2. Cardiac Valves

Valve development is initiated by the formation of EndoMT-derived endocardial cushion cells. Additionally, the endocardial cushions contribute to cardiac septa [38]. EndoMT occurs around E9.5 in the AVC and shortly thereafter in the OFT, followed by valve elongation and stratification post-E14 into leaflets containing three distinct ECM layers: the atrialis/ventricularis (rich in elastin), spongiosa (rich in proteoglycans) and fibrosa (rich in collagens). EndoMT-transformed valve interstitial cells (VICs) populate those leaflets, surrounded by a layer of valve endothelial cells (VECs). Lineage contribution to cardiac valves is complex and differs between AVC and OFT. Immunohistochemistry, electron microscopy and *Tie2-Cre* and *Nfatc1-Cre* mouse lineage tracing experiments demonstrate that AV and SL valve leaflets, as well as AV chordae tendinae and fibrous continuity, are initially generated from endocardial-derived mesenchyme [38,39,40]. The septal leaflets of SL valves however also receive a major contribution from migrating *Wnt1-Cre+* neural crest cells beginning around E10 [41]. SL valves also contain a subset of VICs within the intercalated leaflets that are neither neural crest nor EndoMT-derived [42,43], but were instead reported to be second heart field-derived [42,44]. In addition, epicardial-derived cells significantly contribute to AV valves between E12–E14 [45], populating the parietal leaflets per *Wt1-Cre* lineage tracing [46]. The molecular mechanisms governing valve development are extensive, and will be later discussed in Section 4.2, Section 4.4, and Section 5.1.

### 3.3. Coronary Artery Vasculature

Similar to cardiac valves, the endothelial layer of coronary artery vasculature originates from several distinct lineages. The sinus venosus (SV) is a large embryonic vein that is later incorporated into the right atrium. It undergoes sprouting angiogenesis in mice at E11–E12 to form a subepicardial vascular plexus that vascularizes ventricular myocardium from the outside in. The endocardium, by contrast, contributes to vessels that migrate into the myocardium from the inside out [47,48,49]. In addition, one study found subsets of *Sema3d+* and *Scx+* mouse proepicardial cells (distinct from *Wt1+*/*Tbx18+* populations) that respectively contribute to SV and endocardium and enter the coronary vascular lineage [50].

Lineage tracing identified endocardial cells as a lesser source of coronary artery endothelium. X-gal staining of E11.5 apelin-nlacZ knock-in mouse (expressing β-galactosidase in coronary endothelial cells, but not endocardium) showed an expanding vascular plexus appearing to originate from the SV. This was corroborated by clonal analysis using a VE-Cadherin-*Cre* model, as fluorescence microscopy showed the majority of coronary endothelial clones label sister cells within the SV. The coronary endothelial clones not containing SV sister cells, however, instead contain cells in ventricular or atrial endocardium. Blood island-like structures are also found near the interventricular groove, suggesting that a population of endocardial cells separates from the endocardium to form blood islands before joining the coronary plexus near the interventricular septum [51].

Lineage tracing using an Apelin(*Apj*)-*Cre* mouse line revealed that while SV-derived coronary vessels populate the dorsolateral sides of the heart, they do not populate the ventricular septum or the mid-portion of the ventral face. *Nfatc1-Cre* lineage tracing showed that vessels in these locations instead primarily arise from endocardium. The proepicardium, traced by *Sema3d-Cre*, gives rise to a smaller fraction of vessels spaced uniformly throughout the ventricular walls. Collectively, this indicates that complementary SV- and endocardial-derived migratory routes unite to form the coronary vasculature [47].

The amount of the endocardial contribution to coronary artery endothelium remains subject of debate. Lineage tracing using a *Nfatc1-Cre* knock-in mouse line showed that *Nfatc1+* precursors invade the myocardium at E12.5, forming coronary plexuses at E13.5. Subsequent clonal analysis supported this result and suggested that endocardial cells commit to a coronary endothelial fate right before coronary plexus formation, in a process dependent on myocardial VEGFA to endocardial VEGFR2 signaling [52].

A possible reason for the discrepancy in findings regarding the extent of endocardium contribution is that the *Nfatc1-Cre* line expresses Cre constitutively, and so any transient *Nfatc1* expression in SV or coronary endothelium could lead to overinterpretation of the lineage tracing results [53]. To resolve this dispute, Zhang et al. leveraged *Npr3*, an endocardial specific marker identified via single cell sequencing. Consistent with findings from the *Apj-Cre* mouse, lineage tracing using *Npr3-CreER* showed that ventricular endocardial cells substantially contribute to coronary vessels in the ventricular septum, but do not contribute to the majority of coronary vessels in the embryonic ventricle [54]. Thus, the current consensus is that the majority of non-epicardial-derived coronary vessels in the ventricular free wall are derived from SV, whereas interventricular septal vessels are primarily derived from ventricular endocardium.

Lineage-specific mechanisms regulating coronary endothelium formation have been identified. Loss of *Vegfc* disrupts subepicardial vessel growth emanating from the SV, indicating that it is required for SV-to-coronary migration [47]. ELA-APJ signaling also regulates the SV migratory pathway, as vessel migration onto SV-populated regions is disrupted in *Apj* and *Ela*-deficient mice whereas endocardial-derived areas are unaffected [49]. Interestingly, tracing of *Apj* mutants shows compensatory endocardial response to decreased SV sprouting. *Sox17* is upregulated in endocardial cells when SV angiogenesis is stunted, suggesting it may be a marker of endocardial cells activated to develop into coronary vessels. Areas where endocardial sprouts emerge are hypoxic and positive for HIF-1α as opposed to regions of SV sprouting, suggesting that hypoxia could be an additional physiological cue for endocardial sprouting [49]. However, this is not specific as the medial ventricular wall is also hypoxic prior to epicardial-derived vessel formation [55].

Recently, a subset of intramyocardial coronary arteries were found to form by extension of endocardium-derived vascular tunnels in the neonatal heart in a process implicating Mettl3 and Notch signaling [56]. Interestingly, while endocardium contributes to coronary endothelial cells in the fetal and neonatal heart, *Npr3-CreER* lineage tracing in myocardial infarction-reperfusion, cryoinjury and transverse aortic constriction models has indicated that adult endocardium does not give rise to vascular endothelial cells [57].

### 3.4. Liver Vasculature

The hepatic vasculature is necessary for liver development and repair. Immunostaining of E9.5–E10.5 *Nfatc1*-GFP mice shows that caudal endocardium is located in close proximity to the liver bud, coinciding with hepatic vessel formation [58]. Lineage tracing reveals that descendants of *Nfatc1+* cells are present in liver vasculature, and approximately 40% of the liver vasculature is labeled at E13.5–E15.5. In order to specifically identify the source of contributing endocardium, a strategy comparing *Nfatc1-Dre;Rosa26-RSR-RFP* and *Nfatc1-Dre;Npr3-CreER;Rosa26-RSR-LSL-RFP* (Ai66) embryos was employed. In the case of Ai66, two recombination events (Dre and CreER) are required for RFP expression. As *Npr3-creER* labels atrial and ventricular endocardium but not SV, *Nfatc1-Dre*-mediated recombination alone in the Ai66 reporter does not result in SV labeling. While E10.5 *Nfatc1-Dre;Rosa26-RSR-RFP* embryos show RFP expression in SV and liver, Ai66 embryos do not—thus demonstrating that liver vasculature is derived from *Nfatc1+* SV endocardium but not atrial or ventricular endocardium. *Vegfa* is notably expressed in liver bud while FLK1 protein is expressed in SV endocardium, suggesting that liver endoderm secretion of VEGF-A induces recruitment of FLK1+ endocardial cells through angiogenesis. Ablation of *Flk1* in SV endocardium results in liver hypoplasia and significantly fewer RFP+ cells in mutant liver buds, indicating that VEGF signaling indeed regulates endocardial contribution to liver vasculature [58].

### 3.5. Fat Cells

The epicardium is known to contain fat cells that are epicardial-derived [59], but a distinct subset of fat cells is also found deep within the myocardium. These cells, referred to as intramyocardial adipocytes, are of interest due to association with cardiovascular diseases such as atherosclerosis and arrhythmogenic right ventricular cardiomyopathy. Lineage tracing crossing *Nfatc1-Dre* and *Rosa26-RSR(rox-stop-rox)-RFP* mice reveals that endocardium contributes to intramyocardial adipocytes expressing PPARg, C/EBP and perilipin [60]. This was corroborated using a tamoxifen-inducible *Nfatc1-CreERT2* mouse specifically mapping endocardial cells. By contrast, lineage tracing using *apelin-CreER* in combination with FABP4 staining indicated that coronary vascular endothelial cells do not contribute to adipocytes. Clonal analysis suggested most endocardial cells are capable of differentiating into either coronary vascular endothelial cells or adipocytes, with a minority being bipotent [60]. A more recent study also detected adipocytes that were lineage-traced by *Tie2-Cre* (albeit rarely), further corroborating these findings [61].

### 3.6. Mural Cells

Cardiac pericytes and vascular smooth muscle cells located within coronary vessel walls (referred to as mural cells) primarily arise from epicardial-derived cells that invade the myocardium [62,63,64,65], but endocardium has been shown to contribute as well. Lineage tracing of AVC/OFT PDGFRB+ mesenchymal progenitors in both *Cdh5-CreERT2* and *Tie2-Cre* mice indicates that approximately 20% of mural cells originate from endothelium [66]. Endocardial-derived PDGFRB+/NG2+/PDGFRA- cells from *Nfatc1-CreERT2* mice were closely attached to coronary vessels, but no GFP signal was observed in pericytes of E16.5 *Apln-CreERT2* mice, indicating that endocardial but not subepicardial endothelial cells are a source of mural cells. Further study suggested that migration of PDGFRB+ precursors from the AVC/OFT requires cell autonomous Frizzled 4 and canonical Wnt signaling [66].

A later mouse dual lineage tracing study suggested that developing embryonic endocardium may first contribute to “intermediate” cushion mesenchymal cells, which then migrate into myocardium and differentiate into fibroblasts, coronary mural cells, and adipocytes [67]. The dual tracing approach, which used *Nfatc1-Dre* and *Sox9-CreER*, was necessary since a single Cre recombinase driven by a specific promoter could not specifically label endocardial versus epicardial or neural crest-derived cushion mesenchymal cells.

## 4. Key Endocardial-Myocardial Molecular Pathways

**Figure 3:**
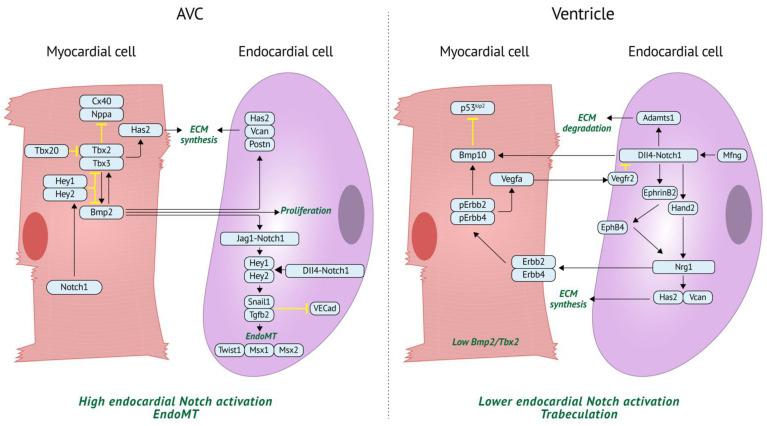
The same signaling pathway (Notch) can produce different effects based on location during early development (E9–E14 in mouse). In the AVC, myocardial cells have high BMP2/TBX2 levels. This represses expression of chamber markers (*Nppa*/*Cx40*) and leads to high activation of the Notch pathway in endocardial cells, resulting in EndoMT, ECM cardiac jelly deposition, and cushion formation. By contrast, *Bmp2*/*Tbx2* is lowly expressed in ventricular chamber myocardium and Notch activation in endocardial cells is lower; thus, EndoMT does not occur. Instead, Notch activates *Nrg1* via *EphrinB2* and *Hand2* to induce trabeculation and expression of *Bmp10* in myocardium. A correct balance between *Nrg1*-regulated ECM synthesis and *Notch1*-regulated ECM degradation is necessary for normal trabeculation to occur. *Vcan*: Versican, *Postn*: Periostin, VECad: VE cadherin, Mfng: Manic fringe, *Nrg1*: Neuregulin.

Key molecular signaling pathway interactions between endocardial and myocardial cells are known to drive heart development. Many of these pathways, such as bone morphogenic protein (BMP) and Notch, are interconnected and can notably drive distinct processes in different cell types depending on spatial location and developmental timepoints (Figure 3).

### 4.1. BMP Regulation of Myocardial Identity and Patterning

BMP signaling plays a crucial role in myocardial patterning and septation in conjunction with T-box transcription factors. BMP2 expression in myocardium is restricted to AVC and OFT regions [68], and *Bmp2* mutant mice fail to specify the AV myocardium [69]. Conditional ablation of *Bmp2* in cardiac progenitors using *Nkx2-5-Cre* (*Bmp2Nkx2.5^del^*) results in cell fate changes in which the heart valve forming region adopts the identity of chamber myocardium [70]. Conversely, overexpression of myocardial BMP2 alone can specify AVC identity, as transgenic ventricular endocardium from an E14.5 *Nkx2.5^Cre/+^;Bmp2^tg/+^* mouse line is EndoMT-competent [71].

*Tbx2* expression overlaps with *Bmp2* and is significantly reduced in *Bmp2* mutants. *Tbx2*-null E9.5 mice exhibit deficient AVC cushion formation and abnormal upregulation of cardiac chamber markers *Nppa*, *Smpx* and *Cx40* [72]. In turn, misexpression of *Tbx2* in chamber myocardium of mice induces ectopic ECM deposition within compact and trabecular myocardium and modifies cardiomyocyte identity to non-chamber type [73]. TBX20 is a direct suppressor of *Tbx2* [74], which attenuates BMP/SMAD-dependent *Tbx2* activation by sequestering SMAD1 and SMAD5 from SMAD4 [71]. It was proposed that from E7.5–E8.5, BMP2 simultaneously induces *Tbx20* and *Tbx2* expression in the cardiac crescent and early heart tube. TBX20 suppression of SMAD1/5 activity then restricts *Tbx2* expression to the developing AVC/OFT with highest levels of BMP signaling, preventing chamber formation and promoting cushion development [75].

### 4.2. BMP Regulation of Valve Development

BMP2 is required for the initiation and maintenance of valve mesenchymal cushion formation [76,77,78]. Addition of BMP2 is sufficient to form cushions in cultured mouse E9.25 AV endocardium even in the absence of myocardium, while exposure to the BMP antagonist noggin inhibits cushion formation even when co-cultured with AV myocardium [79]. Endocardial inactivation of the receptor BMPR1A results in loss of cushion formation, further indicating that BMP2 acts directly on endocardial cells to induce EndoMT [69]. A similar BMP requirement exists in chick embryos [80]. Myocardial BMP2 acts with endocardial TGF-β signaling to directly regulate factors initiating and maintaining EndoMT, including *Twist1*, *Msx1*, and *Msx2* [69]. Consistent with these findings, AVC explants from *Bmp2Nkx2.5^del^* mutant mice cultured in a collagen lattice are unable to undergo EndoMT, and addition of BMP2 rescues the process [70].

TBX2 and TBX3 are redundantly required to maintain *Bmp2* expression. Inactivation of *Tbx2/3* combined or *Bmp2* alone results in very similar phenotypes, including failed AV cushion development and loss of *Tgfb2* and *Has2* gene expression [81]. This indicates a mechanism in which TBX2/3 and BMP2 maintain expression of each other in a feed-forward loop within the AVC. TBX2/3 redundancy may serve to firmly establish the AVC phenotype during early cardiogenesis.

BMP2 induces expression of ECM components, including periostin, versican, and hyaluronan by AV cushion cells [69,82,83]. Failure to deposit ECM results in inability of the endothelium to swell, impairing cushion development [70]. BMP2-induced ECM deposition also regulates cushion expansion and valve maturation. Periostin-null mice exhibit hypoplastic AV valves that fail to completely delaminate and instead contain myocardial tissue. These valves further show decreased organization and collagen expression in addition to chordae tendinae defects. Rescue experiments have substantiated a role for periostin in maturation of cushion mesenchymal cells into mature valve fibroblasts, which is necessary for proper leaflet formation and function [84].

A post-EndoMT role for endocardial-expressed BMP2 has been identified as well. Endocardial-specific *Bmp2* conditional knockout (cKOEndo) mice, generated via crossing of *Bmp2^flox/flox^* and *Nfatc1*^Cre^ mice, exhibit significant reduction in AV cushion size at E13.5 and E16.5, resulting in membranous ventricular septal defects and mitral valve deficiencies. Cushion initiation, proliferation and apoptosis is unaffected in *Bmp2* cKOEndo mice, however, mRNA expression of ECM components (*Vcan*, *Has2*, *Postn*) and critical TFs (*Snail2*, *Twist1*, *Sox9*) was reduced [85].

BMP4 also plays a role in valvulogenesis, as conditional inactivation of *Bmp4* results in mice with significantly smaller AV cushions. Interestingly, these mice do not exhibit defects in the initiation of cushion formation. BMP4 thus appears dispensable for this function, but is required for proper AV septation after cushions have formed [86]. In addition, targeted knockout of anterior heart field-derived myocardial *Bmp4* results in insufficient OFT cushion cells, defective cushion remodeling, and abnormal SL valve formation [87].

### 4.3. BMP Regulation of Trabeculation

*Bmp10* is expressed transiently in ventricular trabecular myocardium from E9.0–E13.5 (a period during which cardiac development shifts from patterning to growth and chamber maturation), and *Bmp10*-null mice exhibit profound hypoplastic ventricular walls and absence of trabeculae. Confocal microscopy of *Bmp10* mutant hearts indicates that BMP10 is not required for either endocardial or myocardial differentiation or the initiation of trabeculation, but is required for further growth of both the ventricular wall and trabeculae [88].

An additional role for BMP2 is evidenced by transgenic E14.5 *Nkx2.5^Cre/+^;Bmp2^tg/+^* mouse embryos exhibiting enlarged trabeculae with increased proliferation. Compact myocardial markers (*Hey2*, *n-Myc*) expanded to trabecular myocardium, suggesting that persistent BMP2 expression maintains cardiomyocytes in a primitive state impairing chamber maturation [71].

### 4.4. Notch Regulation of Valve Development

Notch signaling has been greatly implicated in valve development, as initially indicated by high *notch1b* expression in zebrafish AV valve endocardium coinciding with EndoMT [89,90]. *Notch1* and *Rbpj* mutant mouse embryos exhibit a collapsed endocardium and lack of mesenchymal cushion cells [91]. Constitutive expression of activated *Notch1* (N1IC) in zebrafish embryos leads to enlarged valve size associated with increased mitosis, while treatment with the Notch inhibitor DAPT results in atrophic endocardial cushions. Similar results occur in vitro. Importantly, loss of *Notch1* in mice significantly reduces expression of *Tgfb2* and *Snai1*, resulting in persistent endocardial VE-cadherin expression and failure to initiate EndoMT [91]. Furthermore, deletion of Notch target genes *Hey2* [92,93] or *Hey1*/*Heyl* combined [94] also lead to severe EndoMT defects resulting from decreased *Snail1* and matrix metalloproteinase 2 (*Mmp2*) expression [94].

NOTCH1 is sufficient to activate a pro-mesenchymal gene expression program specifically in endocardial cells. Constitutive ventricular endocardial NOTCH1 activity in E9.5 *Tie2-Cre;N1ICD* mice induces ectopic SNAIL1-dependent EndoMT, conferring “valvular” features to otherwise nonvalvular ventricular endocardium, similarly to BMP2 overexpression phenotypes. Ventricular endocardial cells from these transgenic embryos exhibit migration and expression of mesenchymal markers in collagen gel explant assays, but do not invade the gel matrix. Subsequent addition of BMP2 does promote invasion, indicating that cell detachment and ECM invasion in EndoMT are separately regulated phases [95].

EndoMT induction by BMP2 does not occur in the absence of *Notch1* signaling, whereas ectopic expression of *Bmp2* in ventricular chamber myocardium causes activation of the Notch pathway resulting in EndoMT [96]. In AVC mouse endocardial cells, myocardial BMP2-induced pSmad1/5 directly binds to the *Jag1* promoter, activating the Notch pathway in neighboring endocard cells. Binding of pSmad1/5 to N1ICD further reinforces the Notch-mediated transcription of target genes throughout the AVC endocardium. By contrast in ventricular endocardium, low BMP signaling is insufficient to activate *Jag1* and low levels of pSmad1/5 do not allow for interaction with N1ICD. As a result, EndoMT is not initiated and the endocardium shows chamber-specific features such as *Irx5* expression [96].

DLL4, but not JAG1, specific activation of NOTCH1 is necessary for EndoMT both in vivo and in vitro. However, ablation of endocardial *Jag1* in mice decreases *Hbegf*, resulting in increased proliferation and enlarged valves. Valve development is thus regulated by sequential activation of endocardial NOTCH1, first by DLL4 to induce EndoMT, and then, by JAG1 to limit proliferation [97]. This is corroborated by a further study which stimulated mouse embryonic endocardial cells in vitro with recombinant DLL4 and JAG1, followed by quantitative proteomics analysis of the conditioned media. Notch activation correlated with increased secretion of ECM remodeling and structural proteins and inversely with molecules involved in cell migration. Dll4-Notch signaling contributed to EMT and cell adhesion, whereas *Jag1*-Notch signaling was associated with ECM deposition [98].

### 4.5. Notch Regulation of Trabeculation

Trabeculation is highly regulated by Notch-induced Neuregulin signaling. NRG1 ligand is produced by endocardium, while the receptor *ERBB4* is expressed in myocardial cells that form trabeculae. *Nrg1*-null E10.5 mice show poorly developed trabeculae [99] and both *Erbb2*/*Erbb4*-null mice die prematurely from aborted trabecular development [100,101]. Knockout of *EphrinB2* in mice also results in defects similar to loss of *Nrg1* [102]. Unlike ERBB2/4 however, the EPHB4 receptor is expressed in endocardial cells suggesting an indirect effect of ephrin signaling on myocardium.

Notch activity is highest at presumptive trabecular endocardium and, as development proceeds, concentrates at the base of trabeculae. *Rbpj* and *Notch1* mutant mice show impaired trabeculation and attenuated expression of *Bmp10*, *Nrg1*, and *EphrinB2*. *EphrinB2* itself is a direct Notch target acting upstream of *Nrg1* in the ventricles. Addition of BMP10 to in vitro-cultured *Rbpj* mutants rescues the myocardial proliferative defect, while exogenous NRG1 rescues trabecular differentiation, suggesting that endocardial Notch independently regulates the two processes of cardiomyocyte proliferation and differentiation during trabeculation [103].

NRG1-depleted (*Nrg1^tm/tm^*) mouse embryos show compressed trabeculae and reduced ECM synthesis, whereas Notch-depleted (*Tie2creNotch1^fl/fl^*) embryos exhibit touchdown defects associated with decreased ECM degradation [104]. Treatment of cultured cells with hyaluronidase or metalloprotease inhibitors further indicates that correct ECM dynamics are necessary for endocardial touchdown and ECM bubble formation. Excessive ECM degradation in *Nrg1^tm/tm^* mutants appears to be promoted by the Notch pathway, as ectopic DLL4 and NOTCH1 expression occurs prior to ECM reduction. NRG1 and pERBB2 expression is respectively upregulated in *Tie2creNotch1^fl/fl^* endocardium and myocardium, suggesting that NRG1 contributes to excessive ECM synthesis. Furthermore, *Vegfa* is downregulated in *Nrg1^tm/tm^* embryos and upregulated in *Tie2creNotch1^fl/fl^* embryos. VEGF-A treatment significantly rescues trabeculation in *Nrg1^tm/tm^* embryos, indicating that NRG1-dependent VEGF-A signaling promotes restriction of NOTCH1 activity to touchdown endocardium [104].

Endocardial HAND2, a downstream effector of NOTCH1, is necessary for normal expression of *Nrg1* per mouse *Tie2-Cre*-driven ablation experiments and so regulates specification of trabecular myocardium [105]. Replacement of HAND2 in endocardium via an *Nfatc1-Cre* activatable transgene partly rescues trabeculation and *Nrg1* expression in *Efnb2* cKO mice. Those embryos, however, still show abnormal cardiac phenotype including pericardial edema and hemorrhage, thus indicating that *EphrinB2* signaling has additional HAND2-independent endocardial downstream targets [105].

Heart chamber development has been found to be coordinated through sequential NOTCH1 receptor activation in mouse endocardium, first by endocardial DLL4, and later, by myocardial JAG1/2 signaling. This temporally patterned NOTCH1 activation is determined by the glycosyltransferase manic fringe (MFNG). Early endocardial expression of MFNG promotes DLL4–NOTCH1 interaction and induces trabeculation in the developing ventricle. Subsequent downregulation of MFNG and DLL4 then allows for myocardial JAG1/2–endocardial NOTCH1 interaction, regulating ventricular patterning, maturation, and compaction. Perturbation of this sequence disrupts heart chamber formation [106].

## 5. Endocardial Regulation of Other Cell Types/Processes

### 5.1. Additional Regulation of Valvulogenesis

As previously described, the role of BMP and Notch signaling in valve development has been well characterized. However, other signaling pathways including Hippo and EGFR, transcription factors such as NFATC1 and TBX20, and mechanical cues significantly contribute as well.

Endothelial ablation of *Yap1* in *Tie2-Cre* mice results in reduced proliferation and hypocellular cushions [107]. Collagen gel assays of mutant explants show significantly fewer migrating cells, and *Tie2-Cre* lineage tracing experiments reveal decreased labeling of cells within the AV cushion of mutant embryos, suggesting that endocardial inactivation of *Yap1* may interfere with EndoMT. In vitro siRNA knockdown of *Yap1* decreases expression of *Snail*, *Slug* and *Twist1*, which are known downstream targets of Tgf-β signaling regulated by the Smad2/3/4 complex. Co-IP studies confirms this interaction between YAP1 and SMAD2/3/4, and ChIP-qPCR shows that YAP1 binds to the promoters of *Snail* and *Slug* in response to TGFB1 stimulation, thus modulating and potentiating Tgf-β signaling [107].

Normal cushion formation requires activation of ERBB2 and ERBB3 (but not *ERBB4*) potentiated by hyaluronan. Treatment of *Has2^−/−^* mutant mouse AVC explants with hyaluronan allowed for phosphorylation of ERBB3, rescuing EndoMT impairment [108,109]. The EGFR receptor is particularly important for semilunar valvulogenesis, as both *Egfr^−/−^* and *Egfr^wa2/wa2^* (hypomorphic *Egfr* allele waved-2) mice showed SL valve enlargement from overabundant mesenchymal cells. Compound mutations with *Ptpn11*, which encodes for the protein tyrosine phosphatase SHP2, exacerbated these defects [110].

The GATA family of zinc finger proteins also play a significant role in endocardial cushion development. In particular, endothelial-specific deletion of *Gata4* in mice is embryonic lethal and was found to result in hypocellular cushions associated with decreased *Erbb3* expression [111].

The TF SOX9 is associated with cartilage formation [112] and male sex determination [113], but is also critical for valvulogenesis. *Sox9^flox/flox^;Tie2-cre* mice exhibit hypoplastic endocardial cushions that fail to elongate and form AV valve primordia. *Sox9* expression is additionally maintained during later stages of valve development, and is necessary for maturation [114].

NFATC1 is highly expressed in AVC/OFT endocardium and VECs, and is required for normal valve development, in particular valve elongation [115]. In fact, a subpopulation of mouse valve endocardial cells expressing *Nfatc1* do not undergo EMT, and no cells with high expression of *Nfatc1* were found within cushion mesenchyme. Further study indicated that NFATC1 modulates EndoMT in a cell-autonomous manner by suppressing transcription of *Snail1* and *Snail2* [116]. Thus, the role of NFATC1 is to balance EndoMT versus valve elongation by allocating a sufficient number of cells to the latter.

Endocardial TBX20 is a further regulator of ECM gene expression [117] that is crucial for proliferation, valve elongation, and remodeling [118]. Selective ablation of *Tbx20* using *Tie2-Cre* was found to result in cushion abnormalities, OFT septal defects, and post-E13 prenatal lethality [119]. These mice showed decreased OFT cushion proliferation, impaired post-EndoMT migration, and decreased versican expression. ATAC-seq and ChiP-seq subsequently confirmed binding of TBX20 to an enhancer regulating *Vcan* [119].

Shear stress from blood flow is a significant biomechanical factor regulating valve development [120,121,122]. Hemodynamic quantification studies of the heart showed that the AVC sustained the highest wall shear stress. The main shear-stress responsive rheostat is KLF2, and loss of *Klf2* in the mouse leads to defective cushion formation which is most prominent at the AV canal [123]. Evidence from zebrafish has established that *wnt9b* expression is similarly restricted to the endocardial cells overlying the developing heart valves and is dependent upon both hemodynamic shear forces and *klf2a* expression [121]. Regional differences in blood flow are believed to impact ECM composition during valve maturation, as the atrialis/ventricularis experiences high unidirectional laminar shear stress whereas the fibrosa experiences low bidirectional oscillatory shear stress. Consistent with these findings, *Yap1*, *Klf2* and *Notch1* were specifically found in mice to act in response to mechanosensory cues [124]. In addition, cell-to-cell communication is likely to occur between endothelial and mesenchymal cells. As an example, loss of ADAMTS19, an ECM protein expressed exclusively in valvular interstitial cells, replicates a human valve disease trait in mice and is characterized by upregulation of endothelial *Klf2* expression as evidenced by scRNAseq and immunohistochemistry [125].

Two mechanosensitive pathways have been shown to direct valve cell identity in response to shear stress in zebrafish: transient receptor potential-mediated *klf2a* activation and adenosine triphosphate (ATP)-mediated Ca2+ oscillations leading to *Nfatc1* signaling. Live imaging by Fukui et al. of the fluorescent Ca2+-sensor protein GCaMP7a in zebrafish revealed that endocardial cells displayed Ca2+ oscillations almost exclusively within the valve-forming AVC. As *Nfatc1* is known to be Ca2+-sensitive, they then generated an Nfat binding element reporter line expressing d2EGFP. Nfat reporter expression was observed in AVC endocardial cells, with a similar time course as for the Ca2+ response. These results were further corroborated using a green fluorescent protein (GFP)-*Nfatc1* reporter [126].

In zebrafish, cardiac valve formation is preceded by a symmetry-breaking event promoted by a tissue convergence toward the AVC [127]. This tissue convergence has recently been linked to a global endocardial cell orientation change directed toward the AVC and a cell clustering event that is associated with decreased AVC cell volume. This process was further found to depend on mechanosensitive TRP channels (TRPP2 and TRPV4) and the ECM component hyaluronan [128].

### 5.2. Angiopoietin Regulation of Trabeculation and ECM Metabolism

Angiopoieitin signaling is an important regulator of trabeculation, as the first observable phenotype in *Angpt1*-null mice is alteration of the cardiac trabeculation pattern at E9.5 [129]. TIE2 mediates endothelial angiopoietin signaling, and *Tie2*-deficient mouse embryos die at E10.5 from perturbed vessel organization and trabeculation [130]. Endocardial specific ablation of *Tie2* results in decreased endocardial cell proliferation, impaired migration, and altered expression of N1ICD and *Flk1*. Furthermore, hyperplastic trabeculae resulting from enhanced myocardial proliferation are also noted. This phenotype is associated with upregulation of *Bmp10*, increased retinoic acid signaling, and hyperphosphorylation of ERK1/2 [131]. TIE2 thus appears to regulate trabeculation by two mechanisms: autocrine support of endocardial cell growth and paracrine inhibition of trabecular cardiomyocyte proliferation.

Conversely to BMP2, myocardial ANGPT1 regulates cardiac jelly degradation. *Angpt1*-null mouse embryos exhibit thickened myocardium and abnormally widened space between the endocardium and myocardium filled with cardiac jelly. Excessive versican deposition occurs despite *Vcan* gene expression being unchanged, and cleaved versican is rarely found in mutant embryos, suggesting that loss of *Angpt1* may impede ADAMTS-mediated degradation. Further study indicates that myocardial ANGPT1 activates endocardial *Tie2* to enhance expression of *Adamts1*, promoting cardiac jelly degradation, thus regulating myocardial proliferation and chamber expansion [3].

### 5.3. Conduction System Development

Endocardial cell signaling is required for normal development of the Purkinje fibers of the cardiac conduction system (CCS). A line of transgenic mice in which lacZ is expressed within the embryonic CCS [132] has been used to screen for factors capable of inducing CCS differentiation [133]. This revealed that endocardial NRG1 significantly increases lacZ expression in cardiomyocytes within a specific temporal window of E8.5–E10.5 and causes changes in the electrical activation pattern within the heart, consistent with this ligand playing a critical role in the recruitment of cells to the CCS.

*Etv1* is a downstream target of NRG1 that is expressed in fast conduction tissues and localized to pectinated atrial myocardium (PAM) and ventricular trabecular myocardium. Treatment of E9.5 *Etv1^nlz/+^* mouse hearts with NRG1 expands *Etv1* expression from an exclusively trabecular pattern to a transmural distribution, whereas ERBB2 inhibition causes phenotypic conduction abnormalities and *Etv1* downregulation in PAM and Purkinje cells [134]. This indicates that NRG1 signaling maintains *Etv1* expression. Consistent with knowledge that the Ras-MAPK signaling cascade regulates the transcription of *Etv1*, activated pERBB4, pERBB2, and pERK1/2 are enriched in E13.5 PAM and ventricular trabeculae. Furthermore, postnatal day (P)18 *Etv1*-knockout mice exhibit cardiac abnormalities similar to those from ERBB2 inhibition. *Etv1*-deficient mice also exhibit hypoplasia of the ventricular conduction system from a decrease in Purkinje cells, associated with reduced levels of *Nkx2-5*, *Gja5*, and *Scn5* [134]. ETV1 is therefore required for transducing endocardial NRG1 signals leading to fast conduction gene programming.

During cardiac development, the conduction system in the atrioventricular junction (AVJ) of the heart develops an electrical impulse delay in the AV node that helps ensure effective atrial versus ventricular contraction. Fast conduction markers (*Cx40*, *Nav1.5*) are therefore lowly expressed in AVJ myocardium of HH 10-18 chick hearts [135]. Of particular note, subepicardial ECM deposition and resulting swelling physically separates the endocardial layer of the heart from AVJ myocardium. In two separate experiments, one implanting AVJ myocytes adjacent to cushion endocardium and one exposing myocytes to endocardial-derived paracrine factors, myocardial gene expression and impulse propagation converted to a fast-conducting phenotype [135]. Lack of exposure to endocardial-derived factors (secondary to ECM deposition) is therefore required for a slow conduction fate in the AVJ.

## 6. The Role of Endocardium in Cardiac Disease

Numerous studies suggest an important role for the endocardium in congenital heart disease. Here, we will concentrate on findings from germline sequencing or more functionally oriented studies. For a diverse array of phenotypes, an enrichment of deleterious variations in key endocardial pathways such as VEGF and BMP signaling have been shown, using both candidate and whole-exome approaches. VEGF-A pathway disruption has been implicated in complete atrioventricular canal defect and Down syndrome, and decreased VEGFR2 phosphorylation is associated with tetralogy of Fallot [136]. Although rare, the occurrence of more wide-spread endothelial phenotypes in congenital heart disease with Adams–Oliver syndrome due to NOTCH1 and ARHGAP31 mutations (OMIM 100300, OMIM 616028) provides further evidence of both pleiotropic effects of gene mutations and shared mechanisms of endothelial disease. Perturbation of the Notch cascade has long been recognized as a key driver in congenital heart disease, such as aortic valve disease [137]. Germline mutations of this cascade are responsible for a sizable fraction of left ventricular outflow tract obstruction [138].

### 6.1. Bicuspid Aortic Valve

Bicuspid aortic valve (BAV) is the most common heritable congenital cardiac malformation leading to valve disease, with an increased risk of aortic stenosis and regurgitation. Endocardial loss of *Gata5* in mice results in severe BAV featuring fusion of the right-coronary and non-coronary leaflets, unassociated with alteration in survival or proliferation of mesenchymal cells. GATA5 may instead regulate genes involved in EC migration or differentiation, as a reduction in major TFs including TBX20 and MEF2C is seen in *Gata5*-null mouse hearts. NOTCH1 is linked to BAV [137] and while expression of Notch receptors and *Dll4* is unchanged in *Gata5* mutants, a significant decrease in *Jag1* and *Rbpj*, as well as Notch targets *Nrg1* and *Hey1* is observed. Further study using *Tie2-Cre+Gata5^fl/fl^* mice shows that endocardial GATA5 is required for AV formation [139].

Endothelial NOS (eNOS), also known as nitric oxide synthase 3 (NOS3), has been significantly linked to BAV [140]. Using 3D reconstructions, Peterson et al. were first to report that the non-coronary leaflets of the aortic and pulmonary valves arise from separation of the parietal outflow tract and septal cushions during valve development [44]. Mutant Nos3^−/−^ mice were shown to develop BAV without a raphe as a result of incomplete separation of the parietal outflow tract cushion into the right and non-coronary leaflet. Furthermore, genetic lineage tracing of endothelial, second heart field and neural crest cells revealed altered lineage contribution within the parietal outflow tract cushion of Nos3^−/−^ embryos [44], consistent with prior reporting of a SHF contribution to SL valves [42].

BAV has also been found in over 25% of E14.5 mice mutant for the protocadherin-coding gene *Pcdha9*. SiRNA knockdown of *Pcdh9* causes upregulation of EndoMT-associated genes including *Tgfb1*, *Tgfb2*, *Snail1*, *Notch1* and *Postn*, indicating that protocadherin deficiency can perturb EndoMT and potentially contribute to aortic valve defects [141].

Further discussion on BAV including additional genetic factors is beyond the scope of this article, but specific reviews [142,143,144,145,146] may be referred to for more information.

### 6.2. Mitral Valve Prolapse

Mitral valve prolapse (MVP) is characterized by myxomatous valve degeneration and increased expression of proteoglycan and collagen I. Patient-specific induced pluripotent stem (iPS) cells harboring a mutation in Dachsous (*DCHS1*), associated with MVP [147], recapitulate features of this pathology. Mutant *DCHS1* iPS cells-derived VICs secrete three times as much collagen and six times as much hyaluronan, and overexpress fibrosa and spongiosa ECM proteins, including VCAN, PSTN, APOE, TAGLN, and BGN. SHH signaling is also increased in mutant cells, suggesting that MVP could be rescued by reducing SHH signaling. Indeed, treatment of iPS cells-derived VICs with cyclopamine, a hedgehog pathway inhibitor, together with BMP2 addition at the onset of EMT, reduces ECM protein secretion, while SHH addition increases this [148].

### 6.3. Hypoplastic Left Heart Syndrome

Hypoplastic left heart syndrome (HLHS) is characterized by severe underdevelopment of the LV, mitral valve, aortic valve, and ascending aorta. Emerging evidence from oligogenic mouse models and patient-specific iPSC models suggests that endocardial developmental defects may play a key role in impaired growth of left-sided structures.

First, in a large-scale genetics screen, none of the recovered eight independent HLHS mouse lines had mutations in the same genes, indicating significant heterogeneity [149]. Five of those genes were related to Notch signaling, consistent with prior studies indicating a role for the Notch pathway in the etiology of HLHS [150,151]. In one specific line, *Ohia*, HLHS was associated with mutations in two genes, *Pcdh9* and *Sap130* (sin3A-associated protein 130, a component of the histone deacetylase repressor complex). *Sap130* mutations result in LV hypoplasia, while *Pcdha9* mutations are associated with the aortic hypoplasia and valvular defects in HLHS [149,152].

Second, scRNA sequencing recently identified a role for endocardium in HLHS [153]. scRNA-seq of normal fetal heart revealed unexpectedly high expression of genes previously associated with HLHS [154] in endocardial clusters. Subsequent scRNA-seq of HLHS iPSC-ECs and analysis of HLHS iPSC-derived endocardial endothelial cell (iEEC) lines showed decreased expression of endocardial genes such as *NPR3*. ECM deposition, VEGF signaling and Notch signaling were downregulated in mutant clusters per gene ontology (GO) enrichment analysis. Decreased EndoMT-related gene expression was noted following *TGFB2* stimulation of HLHS iEECs, suggesting EndoMT defects may lead to HLHS. Co-culture assays of iPSC-derived cardiomyocytes (iPSC-CMs) with HLHS iPSC-ECs indicate that HLHS endocard impairs cardiomyocyte growth and maturation. Furthermore, *FN1* (Fibronectin 1) was a top differentially expressed gene in single cell sequencing of both HLHS iEECs and fetal underdeveloped LV. Subsequent siRNA suppression of endocardial *FN1* significantly decreased endocardial and EndoMT genes and impeded iPSC-CM proliferation, while fibronectin addition improved cardiomyocyte proliferation, sarcomere organization and contraction. Reduced *FN1* in HLHS endocardial cells may thus underlie the impaired myocardial development in HLHS [153].

While length constraints do not allow for further discussion in this review, please refer to Table 1 for additional information concerning the genetics of endocardial-related cardiac disease.

## 7. Modeling Endocardial Development and Disease in Stem Cell-Derived Models and Organoids

As evident from the HLHS and MVP studies above, there is a strong need for in vitro models of endocardial-related disease models in order to more efficiently identify potential treatments and regenerative medicine approaches. Stem cell experiments combined with in vivo studies and single cell sequencing demonstrate the potential use of endocardial cells in valve disease modeling. The generation of pure endocardial cell-type populations will allow for separation of endocardial and myocardial signals and potentially a better understanding of primary and secondary causes of endocardium-related heart disease.

A protocol for differentiation of human pluripotent cells into VECs and then VICs was recently developed, showing that hPSCs can recapitulate valvulogenesis [148]. The hiPSC differentiation protocol relies on VEGF and FGF8 to direct a population of hPSC-derived *MESP1*+-sorted cardiac mesoderm progenitors [169] to differentiate towards human pre-valvular endocardial cells (HPVCs). These HPVCs express genes consistent with E9.0 mouse AVC (*TGFB2*, *MSX1*, *ERBB4*). Subsequent treatment with BMP2 induces EndoMT and expression of valve-specific markers including genes typical for fibrosa (*COL1A1*), spongiosa (*VCAN*), and ventricularis (*TAGLN*) layers. *KLF2* is induced by WNT in HPVCs, suggesting the protocol could be used in mechanotransduction experiments [148]. Similarly, WNT induction of HPVC-derived VICs leads to increased ADAMTS19 expression, which is necessary for normal valve development [125]. A modified protocol using prolonged treatment with VEGF, TGFB1, and BMP4 for induction of valvular endothelial cells has been recently proposed [170].

Novel factors promoting endocardial development can be identified via in vitro differentiation studies, as in a recent example implicating BMP10. When combined with bFGF (FGF2), BMP10 increases the proportion of hPSC-derived NKX2-5+/CD31+ cells (representing the earliest stage of endocardial development) from a transgenic *HES3-NKX2-5eGFP* line [171]. Trabecular markers (*NPPA/B*) are significantly upregulated in co-cultured target cardiomyocytes, indicating that endocardial-like hPSC-derived NKX2-5+CD31+ cells are capable of inducing a trabecular fate. Furthermore, NKX2-5+CD31+ cells treated with BMP2/bFGF/*TGFB2* can undergo EndoMT giving rise to CD31–PDGFR-B+ mesenchymal cells expressing VIC markers (*PRRX2*, *TIMP3*, *NR4A2*). However, consistent with these observations and the fact that *Bmp10* is highly expressed in mouse ventricular, but not valve, endocard, it may be possible that this protocol yields *NPR3+* trabecular endocardium, which was shown to undergo EndoMT and form VIC-like cells upon stimulation by BMP2 [96]. The study’s protocol may nevertheless assist in future modeling of human heart development and endocardium-related disease in vitro [171].

Self-organized three-dimensional cellular structures termed organoids are increasingly being used as models to recapitulate human development. Recent progress has been made in generating organoid cardiac tissues [172,173,174]. Lewis-Israeli et al. generated human heart organoids using Wnt signaling modulation that were transcriptomically, structurally and cellularly comparable to fetal cardiac tissue. This platform was further shown to be capable of modeling gestational diabetes-induced congenital heart defects [172]. Hofbauer et al. established self-organizing cardioids that formed chamber-like structures containing a cavity, a process governed by Wnt-BMP signaling requiring HAND1. Upon cryoinjury, these cardioids showed significant ECM accumulation, known to be an early sign of regeneration and heart disease [173]. In the future, further improvement in generating cardiac organoids should allow for study of endocardial-related diseases. The described techniques allow for the possibility of manipulating gene pathways and cellular contributions to organ formation, which will in turn contribute to understanding of disease etiology.

## 8. Conclusions

The endocardium is a complex and highly plastic cell type that has historically been less investigated. Significant progress has however been made in unraveling the sources and future fates of endocardial cells. As underscored by Miao et al.’s HLHS study, an increased understanding over the coming years of the molecular pathways and factors involved, leveraging techniques such as single cell sequencing, is desirable and highly likely to be fruitful in both translational and regenerative medicine.

## Figures and Tables

**Table 1:** Endocardial-related cardiac diseases.

Cardiac Disease	Implicated Genes	Reference
Bicuspid aortic valve (BAV)	NOTCH1 GATA5 NOS3 PCDHA9	Garg et al. [137] Laforest et al. [139] Lee et al. [140] Liu et al. [149], Yagi et al. [152]
Mitral valve prolapse (MVP)	FLNA *DCHS1*	Kyndt et al. [155] Durst et al. [147]
Hypoplastic left heart syndrome (HLHS)	NOTCH1 PCDHA9 + SAP130 *FN1*	Iascone et al. [150], Theis et al. [151] Liu et al. [149], Yagi et al. [152] Miao et al. [153]
Tetralogy of Fallot (TOF)	GATA4 NKX2-5 JAG1 TBX5 FLT4	Zhang et al. [156] Yang et al. [157] Schott et al. [158] Bauer et al. [159] Baban et al. [160] Jin et al. [154]
Left ventricular non-compaction syndrome (LVNC)	MYH7, ACTC, TNNT2 MYBPC3, TNNI3, TPM1	Klaassen et al. [161] Hoedemaekers et al. [162]
Aortic stenosis (AS)	ELN ADAMTS19	Curran et al. [163] Wünnemann et al. [125]
Aortic dilation (AD)	ACTA2	Guo et al. [164], Yetman et al. [165]
Complete AV canal defect (CAVC)	VEGFA NFR2F2	Ackerman et al. [136] Al Turki et al. [166]
Adams-Oliver Syndrome (AOS)	NOTCH1 ARHGAP31	Stittrich et al. [167] Southgate et al. [168]
Left ventricular outflow tract obstruction (LVOTO)	NOTCH1, ARHGAP31	Preuss et al. [138]

## Data Availability

Not applicable.
